# Age-Related Differences in the Time Course of Coagulation and Fibrinolytic Parameters in Patients with Traumatic Brain Injury

**DOI:** 10.3390/ijms21165613

**Published:** 2020-08-05

**Authors:** Ryuta Nakae, Yu Fujiki, Yasuhiro Takayama, Takahiro Kanaya, Yutaka Igarashi, Go Suzuki, Yasutaka Naoe, Shoji Yokobori

**Affiliations:** 1 Department of Emergency and Critical Care Medicine, Nippon Medical School, 1-1-5, Sendagi, Bunkyo-ku, Tokyo 113-8603, Japan; ccm2199@yahoo.co.jp (Y.T.); t-kanaya@nms.ac.jp (T.K.); igarashiy@nms.ac.jp (Y.I.); shoji@nms.ac.jp (S.Y.); 2 Emergency and Critical Care Center, Kawaguchi Municipal Medical Center, 180, Nishiaraijuku, Kawaguchi-shi, Saitama 333-0833, Japan; siam1999@nms.ac.jp (Y.F.); g.suzuki417@gmail.com (G.S.); ynaoe1120@mac.com (Y.N.)

**Keywords:** brain injuries, traumatic, aged, middle aged, blood coagulation disorders, fibrinogen, fibrin fibrinogen degradation products

## Abstract

Coagulopathy and older age are common and well-recognized risk factors for poorer outcomes in traumatic brain injury (TBI) patients; however, the relationships between coagulopathy and age remain unclear. We hypothesized that coagulation/fibrinolytic abnormalities are more pronounced in older patients and may be a factor in poorer outcomes. We retrospectively evaluated severe TBI cases in which fibrinogen and D-dimer were measured on arrival and 3–6 h after injury. Propensity score-matched analyses were performed to adjust baseline characteristics between older patients (the “elderly group,” aged ≥75 y) and younger patients (the “non-elderly group,” aged 16–74 y). A total of 1294 cases (elderly group: 395, non-elderly group: 899) were assessed, and propensity score matching created a matched cohort of 324 pairs. Fibrinogen on admission, the degree of reduction in fibrinogen between admission and 3–6 h post-injury, and D-dimer levels between admission and 3–6 h post-injury were significantly more abnormal in the elderly group than in the non-elderly group. On multivariate logistic regression analysis, independent risk factors for poor prognosis included low fibrinogen and high D-dimer levels on admission. Posttraumatic coagulation and fibrinolytic abnormalities are more severe in older patients, and fibrinogen and D-dimer abnormalities are negative predictive factors.

## 1. Introduction

Traumatic brain injury (TBI) remains a leading cause of trauma death and will overtake many other disorders as a major cause of death and disability by 2020 [1]. However, our understanding of the nature of this condition and its management is not definitive [2]. A large meta-analysis of closed TBI patients with Glasgow Coma Scale (GCS) scores of 3–8 [3] found that older age was consistently associated with a worse outcome after TBI, and mortality and unfavorable outcomes occurred in 52% and 74%, respectively, of affected individuals aged >55 y. TBI is commonly associated with acute perturbations of coagulation and fibrinolysis. In a recent meta-analysis [4,5], the overall prevalence of TBI-associated coagulopathy was 32.7–35.2%, and a strong association was seen between abnormal hemostasis and a poor outcome. Similarly, a major trauma registry study [6] in Germany reported that 22.7% of patients with isolated TBI presented with an acute coagulopathy on admission to the emergency department, which was associated with increased rates of morbidity and mortality. Nevertheless, the mechanisms behind the association of coagulopathy with age in the acute phase of TBI are unclear. We speculated that acute posttraumatic coagulation/fibrinolytic changes would be more severe in older TBI patients, and that these changes may explain, in part, why elderly TBI patients have a poorer outcome. To evaluate this hypothesis, we investigated the association between age and coagulation/fibrinolytic parameters, including prothrombin time (PT)-international normalized ratio (INR), activated partial thromboplastin time (APTT), fibrinogen concentration, and plasma levels of D-dimer, which is the primary degradation product of cross-linked fibrin, within the first 3–6 h after injury.

## 2. Results

### 2.1. Case Selection

**Figure 1:**
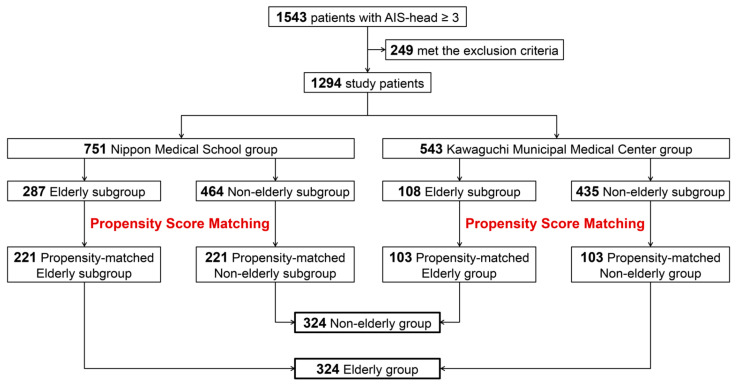
Case selection. Patients diagnosed with severe traumatic brain injury (TBI), defined as Abbreviated Injury Score (AIS)-head ≥3, with initial blood samples obtained ≤1 h after injury, were eligible. Exclusion criteria were an initial blood sample drawn >1 h after injury, incomplete information on time of injury, absent coagulation/fibrinolytic parameter measurements between 3 and 6 h after injury, age <16 y, presence of infection, liver failure, hematological disease, pregnancy, hypotension (systolic blood pressure <90 mmHg) or hypoxemia (PaO_2_ < 60 mmHg) at admission, malignancy, use of anticoagulant or antiplatelet agents, cardiopulmonary arrest prior to or on arrival in the hospital, death as a result of non-TBI conditions, and incomplete information on outcomes at 3 months after injury. Elderly group, patients aged ≥75 y; non-elderly group, patients aged 16–74 y.

We evaluated 1543 cases with TBI managed from April 2007 to December 2018. Of these, 345, 405, and 523 cases (some of the cases overlap) were from our previous studies published in 2016, 2017, and 2019, respectively [7,8,9]. The mechanisms of injury were traffic accidents in 899 patients (58.3%), falls in 564 patients (36.5%), and other mechanisms in 80 patients (5.2%). A total of 1294 cases met the inclusion criteria and were enrolled in the study. The patients were divided into two groups based on whether they were admitted to Nippon Medical School (“Nippon Medical School group”) or Kawaguchi Municipal Medical Center (“Kawaguchi Municipal Medical Center group”). Each group was further divided into two subgroups according to the definition of elderly of the Joint Committee of the Japan Gerontological Society and the Japan Geriatrics Society [10], namely those aged ≥75 y (“elderly subgroup”) and 16–74 y (“non-elderly subgroup”) (Figure 1).

Propensity score matching to adjust for baseline characteristics between the subgroups created a matched cohort of 221 pairs in Nippon Medical School group and 103 pairs in Kawaguchi Municipal Medical Center group for a total of 324 pairs (Figure 1). Table 1; Table 2 show the baseline characteristics of the unmatched and propensity score-matched subgroups from Nippon Medical School and Kawaguchi Municipal Medical Center, respectively. The elderly group had a higher percentage of acute subdural hematoma (ASDH) according to comparisons between unmatched groups. On propensity score matching, we found that the matched patients were finely balanced with regard to characteristics, showing a standardized mean difference of <0.1 between groups.

### 2.2. Relationship between Coagulation/Fibrinolytic Parameters and Age

#### 2.2.1. PT-INR

**Figure 2:**
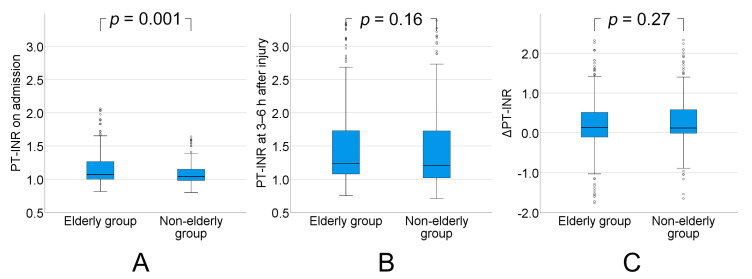
(**A**) Prothrombin time-international normalized ratio (PT-INR) on admission was significantly higher in the elderly group (aged ≥75 y) than in the non-elderly group (aged 16–74 y) (*p* = 0.001). (**B**) PT-INR increased from admission to 3–6 h after injury in both groups, but there was no significant difference in PT-INR at 3–6 h after injury between the groups (*p* = 0.16). (**C**) There was no significant difference in the increase in PT-INR between admission and 3–6 h after injury between the groups (*p* = 0.27). ΔPT-INR = PT-INR at 3–6 h−PT-INR on admission.

The PT-INR (normal range: 0.8–1.2) on admission was significantly higher in the elderly group than in the non-elderly group (median: 1.07 (interquartile range (IQR): 1.00–1.27) vs. median: 1.04 (IQR: 0.98–1.15), *p* = 0.001). The PT-INR increased from admission to 3–6 h after injury in both groups, with no significant differences in PT-INR at 3–6 h after injury (median: 1.24 (IQR: 1.08–1.74) vs. median: 1.21 (IQR: 1.02–1.73), *p* = 0.16) or in the increase in PT-INR between admission and 3–6 h after injury (median: 0.14 (IQR: −0.11–0.51) vs. median: 0.12 (IQR: −0.01–0.58), *p* = 0.27) between the groups (Figure 2).

#### 2.2.2. APTT

**Figure 3:**
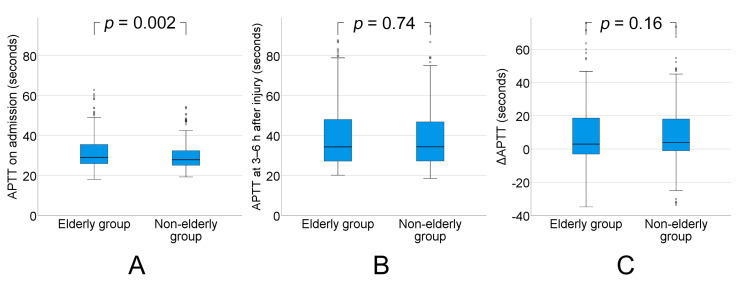
(**A**) Activated partial thromboplastin time (APTT) on admission was significantly higher in the elderly group (aged ≥75 y) than in the non-elderly group (aged 16–74 y) (*p* = 0.002). (**B**) APTT increased from admission to 3–6 h after injury in both groups, but there was no significant difference in APTT at 3–6 h after injury between the groups (*p* = 0.74). (**C**) There was no significant difference in the increase in APTT between admission and 3–6 h after injury between the groups (*p* = 0.16). ΔAPTT = APTT at 3–6 h−APTT on admission.

The APTT (normal range: 24–36 s) on admission was significantly higher in the elderly group than in the non-elderly group (median: 29.1 (IQR: 25.9–35.7) vs. median: 27.9 (IQR: 25.1–32.5), *p* = 0.002). The APTT increased from admission to 3–6 h after injury in both groups, but, again, there were no significant differences in APTT at 3–6 h after injury (median: 34.4 (IQR: 27.2–48.1) vs. median: 34.4 (IQR: 27.3–47.0), *p* = 0.74) and the increase in APTT between admission and 3–6 h after injury (median: 2.98 (IQR: −3.06–18.65) vs. median: 4.0 (IQR: −1.00–18.07), *p* = 0.16) between the groups (Figure 3).

#### 2.2.3. Fibrinogen

**Figure 4:**
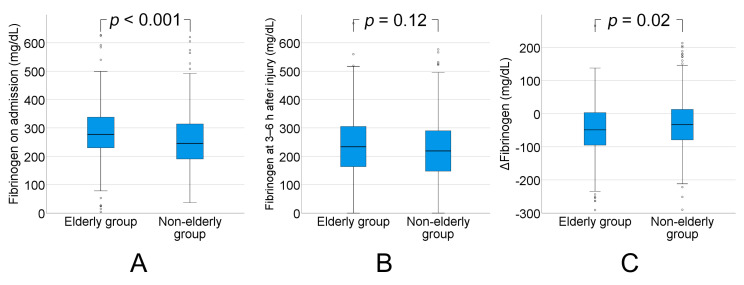
(**A**) Fibrinogen concentration on admission was significantly higher in the elderly group (aged ≥75 y) than in the non-elderly group (aged 16–74 y) (*p* < 0.001). (**B**) Fibrinogen concentration decreased from admission to 3–6 h after injury in both groups, but there was no significant difference in fibrinogen concentration at 3–6 h after injury between the groups (*p* = 0.12). (**C**) The reduction in fibrinogen concentration between admission and 3–6 h after injury was significantly higher in the elderly group than in the non-elderly group (*p* = 0.02). ΔFibrinogen = fibrinogen at 3–6 h−fibrinogen on admission.

Fibrinogen concentration (normal range: 200–400 mg/dL) on admission was significantly higher in the elderly group than in the non-elderly group (median: 277 (IQR: 230–338) vs. median: 246 (IQR: 190–314), *p* < 0.001). Fibrinogen concentration decreased from admission to 3–6 h after injury in both groups, but there was no significant difference in fibrinogen concentrations at 3–6 h after injury between the groups (median: 234 (IQR: 163–306) vs. median: 219 (IQR: 147–291), *p* = 0.12). The reduction in fibrinogen concentration between admission and 3–6 h after injury was significantly higher in the elderly group than in the non-elderly group (median: −49 (IQR: −96–4) vs. median: −33 (IQR: −79–13), *p* = 0.02) (Figure 4).

#### 2.2.4. D-dimer

**Figure 5:**
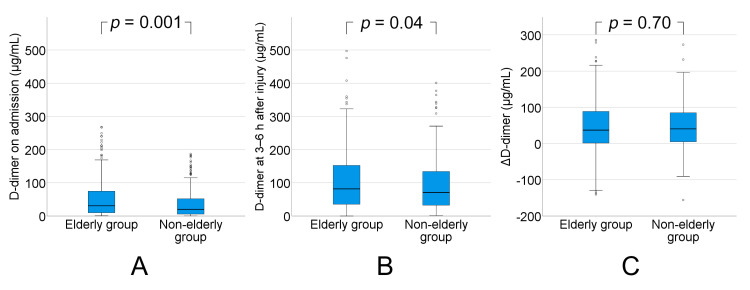
(**A**) Plasma levels of D-dimer on admission were significantly higher in the elderly group (aged ≥75 y) than in the non-elderly group (aged 16–74 y) (*p* = 0.001). (**B**) Plasma levels of D-dimer increased from admission to 3–6 h after injury in both groups, and the levels at 3–6 h after injury was also significantly higher in the elderly group than in the non-elderly group (*p* = 0.04). (**C**) There was no significant difference in the increase in plasma levels of D-dimer between admission and 3–6 h after injury between the groups (*p* = 0.70). ΔD-dimer = D-dimer at 3–6 h−D-dimer on admission.

Plasma levels of D-dimer (normal range: 0.0–1.0 μg/mL) on admission was significantly higher in the elderly group than in the non-elderly group (median: 30.7 (IQR: 9.9–74.6) vs. median: 19.9 (IQR: 5.7–51.8), *p* = 0.001). Plasma levels of D-dimer increased from admission to 3–6 h after injury in both groups, and the levels at 3–6 h after injury were also significantly higher in the elderly group than in the non-elderly group (median: 81.6 (IQR: 35.2–152.5) vs. median: 70.7 (IQR: 32.5–134.0), *p* = 0.04). There was no significant difference in the increase in plasma levels of D-dimer between admission and 3–6 h after injury between the groups (median: 37.1 (IQR: 1.3–89.7) vs. median: 40.4 (IQR: 4.9–84.9), *p* = 0.70) (Figure 5).

#### 2.2.5. Correlation between Age and Outcome

The Glasgow Outcome Scale (GOS) scores at 3 months post-injury in the elderly group were significantly lower than those in the non-elderly group (*p* = 0.004) (Table 3).

#### 2.2.6. Independent Risk Factors for Poor Prognosis at the Time of Admission

On multivariate logistic regression analysis that included initial variables, independent risk factors for poor prognosis at the time of admission included older age (*p* < 0.001), low GCS score (*p* < 0.001), high intracranial Abbreviated Injury Score (AIS-head) (*p* < 0.001), high injury severity score (ISS) (*p* < 0.001), low fibrinogen level (*p* = 0.001), and high D-dimer level (*p* = 0.006) (Table 4).

## 3. Discussion

In this analysis of the time course of coagulation/fibrinolytic parameters in the acute phase of TBI, we found that coagulation parameters such as PT-INR, APTT, and fibrinogen at admission, and fibrinolytic parameters such as D-dimer at admission and 3–6 h after injury differed between the elderly and non-elderly groups. The reduction in fibrinogen concentration between admission and 3–6 h after injury was significantly higher in the elderly group than in the non-elderly group. Low fibrinogen levels and high D-dimer levels at admission were independent risk factors for poor prognosis.

Several mechanisms are suggested to explain the pathophysiology underlying TBI-induced coagulopathy. These include tissue factor (TF) activation and hyperfibrinolysis.

Keimowitz et al. [11] and Goodnight et al. [12] first proposed that injured brain tissue releases TF into the systemic circulation, and that this, in turn, causes coagulopathy. TF is highly expressed in the central nervous system [13] but is usually excluded from the systemic circulation due to the blood–brain barrier, and not in direct contact with coagulation factors. Direct vessel injury or fragmentation from microvascular failure leads to intravascular release of TF and can activate the extrinsic coagulation pathway. This results in thrombin formation in the initiation phase of coagulation, followed by platelet dysfunction and exhaustion [14,15]. Coagulopathy then leads to fibrin deposition and intravascular microthrombosis, and possibly also posttraumatic cerebral infarction [16,17]. This might further increase the consumption of coagulation factors, which could potentially induce further bleeding.

Hyperfibrinolysis is also considered to cause coagulopathy after TBI. The constantly active fibrinolytic system works to prevent inappropriate thrombus formation and may be induced as part of a negative feedback loop within the hemostatic cascade. Plasmin is the cleavage product of circulating plasminogen, and is the primary effector of fibrinolysis. Further upstream, tissue plasminogen activator together with urokinase plasminogen activator represent the primary activators of plasminogen. Plasmin itself is rapidly inactivated by α2 plasmin inhibitor (α2-PI) to make the plasmin-α2-PI complex [18]. Kushimoto et al. [19] indicated that the depletion of α2-PI and associated increase in plasmin might contribute to hyperfibrinolysis and produce a bleeding diathesis as a result of broad activation, or via the dissolution of a newly developed fibrin clot locally within the damaged brain. In the present study, increases in PT-INR and APTT, and decreases in fibrinogen concentration from admission to 3–6 h after injury, were demonstrated. Prolonged PT-INR and APTT are produced by activation of the coagulation pathway and depletion or dysfunction (or both) of plasma coagulation factors as a result of TBI. The reduction in plasma fibrinogen concentration might be attributed to consumption to make a fibrin clot, or to direct destruction. If the D-dimer level is elevated, increased plasmin activity secondary to enhanced fibrinolytic activity promotes fibrin degradation; that is, both hypercoagulability and hyperfibrinolysis are recognized in the acute phase of TBI. Particularly, D-dimer activity was markedly increased in the elderly group both on admission and 3–6 h after injury, as can be seen in Figure 5. Hyperfibrinolysis can produce hemorrhage expansion via the degradation of coagulation factors, or breakdown of formed fibrin clots, as well as impaired clot formation resulting from excessive generation of fibrin degradation products [5,20,21,22], and is associated with a poorer outcome.

Several studies have demonstrated that fibrinogen concentration increases with age [23,24,25,26]. Hager et al. [24] showed that fibrinogen concentration increased by 25 mg/dL per decade, thereby reaching concentrations above 320 mg/dL in 81% of those older than 65 y. An increase in fibrinogen might reflect a multitude of processes, including acute phase reactions, as well as risk factors, subclinical degeneration of vascular endothelium, or the more frequent activation of coagulation and fibrinolysis, in which interleukin 6 is a potential mediator [24,27]. Similarly, in the present study, the fibrinogen concentration on admission was significantly higher in the elderly group than in the non-elderly group. Interestingly, the degree of reduction in fibrinogen concentration between admission and 3–6 h after injury was significantly higher in the elderly group than in the non-elderly group. A higher consumption of fibrinogen may be one of the reasons that elderly TBI patients have a poorer outcome.

The most marked differences were detected in the plasma levels of D-dimer between the groups in the present study. Several reports have shown that older people have increased baseline D-dimer levels [28,29,30,31,32]. Cadroy et al. [28] showed that the mean plasma levels of D-dimer were two- to five-fold higher in subjects with age ≥60 y as compared to those <60 y (0.033–0.433 vs. 0.312–1.180 μg/mL). Hager et al. [29] demonstrated that a higher D-dimer may be due to changes in fibrinogen catabolism, which were about 40% higher in elderly subjects. Tita-Nwa et al. [32] also demonstrated that the increasing levels of D-dimer with age are due to a mild pro-inflammatory state and elevated levels of co-morbidities given that erythrocyte sedimentation rate, a marker of systemic inflammation, has been independently associated with elevated D-dimer levels. Pieper et al. [31] showed that the higher D-dimer levels in the elderly can be explained by increased production of TF as a response to given levels of cytokine stimulus from endothelial cells. Nevertheless, the association between increased plasma D-dimer and age after TBI is unclear. We identified the onset of hyperfibrinolysis within 1 h after injury, and found that this change is signaled by elevated plasma levels of D-dimer. Yokota et al. [33] reported that worse outcomes following TBI in older patients were associated with the activation of cerebral endothelium, as shown by higher serum levels of thrombomodulin and von Willebrand factor than in young and middle-aged patients. These authors [34] also showed that von Willebrand factor levels paralleled those of plasma fibrinogen degradation products. Johansson et al. [35] also demonstrated that a high syndecan-1 level, which is a marker of endothelial glycocalyx degradation, on admission is related to fibrinolysis, as well as to higher mortality in trauma patients. These results provide evidence for the idea that the fibrinolytic cascade is more frequently activated in older TBI patients due to the raised sensitivity of cerebral endothelial cells to trauma.

Several recent papers have reported the time-course of coagulation and fibrinolytic variables in TBI in its acute phase [7,9,36,37]. We reported that the plasma levels of D-dimer on admission were higher in TBI patients with a poor outcome than those with a good outcome [7]. Furthermore, we also showed that the plasma levels of D-dimer were higher in TBI patients aged >55 y than those aged 16–55 y [9]. In both studies, however, there were no differences in fibrinogen concentration on admission between patients with a good outcome and those with a poor outcome, and between patients aged > 55y and those aged 16–55 y. We consider that this is due to the fact that patients receiving fresh frozen plasma (FFP) transfusions containing fibrinogen were excluded in these studies to remove their impact on coagulation/fibrinolytic parameters. In the present study, propensity score-matched analysis allowed for a more accurate determination of fibrinogen hemodynamics. In addition, our present study differs from these studies in that it included patients with multiple trauma. Consequently, in the present study, high D-dimer levels at admission and the reduction in fibrinogen concentration in the acute phase of TBI were noted in the elderly group. To summarize, the characteristics of elderly TBI patients included the rapid consumption of coagulation factors, leading to fibrinolysis. This may be a factor associated with their poorer prognosis. Future studies should investigate whether the early identification of acute coagulopathy and prevention of delayed hemostatic perturbation may be associated with better morbidity and mortality outcomes in aged TBI patients.

This study has several limitations. First, this is a retrospective study. Further large prospective studies are needed to validate our results. Second, different reagents were used in coagulation/fibrinolytic parameter measurements at each institution. To avoid the effects of reagent differences, the patients were divided into two groups based on study institution, and propensity score-matched analyses were performed in each group. Third, the differences in the mechanisms of TBI between Japan and other countries may be due to the higher average age of the Japanese population. As a result, severe TBI in the elderly may be more common. Finally, measurement of the GOS score at 3 months may be somewhat early in patients with severe TBI. In addition, the GOS-Extended, not GOS, may be needed to assess the outcome of TBI patients accurately. However, we were only able to collect outcome data of many patients from the GOS at 3 months post-injury. Additional investigation with a longer-term follow-up of recovery after severe TBI in these patients is warranted.

## 4. Materials and Methods

### 4.1. Patient Population

We retrospectively investigated demographic, clinical, and radiologic findings from 1543 consecutive patients with TBI admitted to the Critical Care Center of Nippon Medical School and Kawaguchi Municipal Medical Center from April 2007 to December 2018. Of these, 345, 405, and 523 cases were (some of the cases overlap) from our previous studies published in 2016, 2017 and 2019, respectively [7,8,9]. Patients diagnosed with severe TBI, defined previously as AIS-head ≥3 [4,5,7], with initial blood samples obtained ≤1 h after injury, were eligible. Diagnosis was determined from findings of computed tomography (CT) and magnetic resonance imaging (MRI), after independent evaluation by study intensivists and neurointensivists of intracranial and extracranial AIS, CT, and MRI scans. Exclusion criteria were an initial blood sample >1 h after injury, incomplete information on time of injury, absent coagulation/fibrinolytic parameter measurements between 3 and 6 h after injury, age <16 y, presence of infection, liver failure, hematological disease, pregnancy, hypotension (systolic blood pressure <90 mmHg) or hypoxemia (PaO_2_ < 60 mmHg) at admission, malignancy, use of anticoagulant or antiplatelet agents, cardiopulmonary arrest prior to or on arrival in the hospital, death as a result of non-TBI conditions, and incomplete information on outcomes at 3 months after injury. The study was approved by our Institutional Review Boards (Nippon Medical School: #30-09-999, 12 November, 2018 and Kawaguchi Municipal Medical Center: #2018-27, 20 November, 2018).

We collected data on patient age; sex; GCS score at admission; AIS-head, face, chest, abdomen, extremities, and external [38]; ISS [39]; and the volume of FFP administered. In all patients, blood samples for the initial (within 1 h after injury) PT-INR, APTT, and plasma levels of fibrinogen and D-dimer were drawn on arrival at the Emergency Department. Tests were routinely repeated between 3 and 6 h after injury. CT scans and MR images on admission and at follow-up were independently evaluated, and the type of head injury was classified using radiologic findings as ASDH, acute epidural hematoma (AEDH), traumatic intracerebral hematoma/contusion (TICH), and traumatic subarachnoid hemorrhage (TSAH) (some patients had more than one diagnosis).

### 4.2. Management of TBI

Treatment was provided immediately on arrival at the emergency department based on guidelines for the management of TBI produced by the Japan Society of Neurotraumatology [40,41]. All patients underwent brain CT after detailed neurological workup and initial resuscitation. In most cases, a 2nd CT scan was obtained within 3 h after admission, and again whenever clinical deterioration or indications of elevated intracranial pressure were seen. When CT revealed no significant abnormality but TBI was still suspected, an MRI was immediately conducted.

### 4.3. Assay of Coagulation/Fibrinolytic Parameters

Blood samples were obtained in ethylenediaminetetraacetic acid (EDTA) plasma and citrate. PT was measured using the coagulating time method (Nippon Medical School: Coagpia^®^ PT-N, Sekisui Medical Corp., Tokyo, Japan; Kawaguchi Municipal Medical Center: Dade Innovin^®^, Sysmex Corp., Kobe, Japan). The APTT was measured using the coagulating time method (Nippon Medical School: Coagpia^®^ APPT-N, Sekisui Medical Corp., Tokyo, Japan; Kawaguchi Municipal Medical Center: Thrombocheck APTT-SLA^®^, Sysmex Corp., Kobe, Japan). Fibrinogen was measured using the thrombin coagulating time method (Nippon Medical School: Coagpia^®^ Fbg, Sekisui Medical Corp., Tokyo, Japan; Kawaguchi Municipal Medical Center: Thrombocheck Fib (L)^®^, Sysmex Corp., Kobe, Japan). D-dimer was measured using the latex immunoassay method (Nippon Medical School: Nanopia^®^ D-dimer, Sekisui Medical Corp., Tokyo, Japan; Kawaguchi Municipal Medical Center: LIAS Auto D-dimer Neo^®^, Sysmex Corp., Kobe, Japan).

### 4.4. Statistical Analysis

Data are expressed as number (%) or median (IQR). Continuous variables were compared between groups using Student’s *t*-test or the Mann–Whitney *U*-test, and categorical variables were compared using the *χ^2^* test. A value of *p* < 0.05 was considered statistically significant. All statistical analyses were performed using commercial software (SPSS Version 25.0^®^; IBM Corp., Armonk NY, USA).

#### 4.4.1. Propensity Score-Matched Analysis

First, the patients were divided into 2 groups based on whether they were admitted to Nippon Medical School or Kawaguchi Municipal Medical Center. This process was necessary because different reagents were used in coagulation/fibrinolytic parameter measurements at each institution. Second, in each group, we performed one-to-one matching analysis between the elderly subgroup (aged ≥75 y) and the non-elderly subgroup (aged 16–74 y), based on estimated propensity scores [42] for each patient to adjust baseline characteristics between the subgroups. The rationale of 75 y as a cutoff point was based on the definition of “elderly” by the Joint Committee of the Japan Gerontological Society and the Japan Geriatrics Society [10]. We assessed the propensity score by fitting a logistic regression model as a function of the patients’ demographic and clinical characteristics, including the following, which were previously reported to have the potential to affect coagulation/fibrinolytic parameters and the outcome in patients with severe TBI: Age [3,6,7]; sex; GCS score at admission [6,7,37]; the presence of ASDH, AEDH, TICH, and TSAH; AIS-head, face, chest, abdomen, extremities, and external [6,7,37,43,44]; ISS [6,37,43,44]; and the volume of FFP [8,45,46]. We evaluated the balance in baseline variables using standardized differences, in which an absolute value of <10% was considered as balanced [42].

#### 4.4.2. Multivariate Logistic Regression Analysis

Multivariate logistic regression analysis (forced entry method) was performed to identify risk factors for poor prognosis at the time of admission [47,48]. The explanatory variables, which were previously reported to have the potential to affect outcome, included age [3,6,7], GCS score at admission [6,7,37], the presence of ASDH, AEDH, TICH, and TSAH, AIS-head [6,7,37], ISS [6,37,43,44], and coagulation/fibrinolytic parameters such as initial PT-INR, APTT, and plasma levels of fibrinogen and D-dimer. The response variable was a good outcome or poor outcome at 3 months post-injury. The good outcome included patients with good recovery or moderate disability (GOS score = 5 or 4, respectively) and the poor outcome included severe disability, vegetative state, or death (GOS score = 3, 2, or 1, respectively) [49]. The GOS was independently evaluated by study neurointensivists using in-person contact or telephone and mail communications to the hospital where patients were transferred from our hospital after discharge.

## 5. Conclusions

The present study using propensity score-matched analysis revealed that consumption of fibrinogen and fibrinolytic abnormalities is more severe in aged TBI patients during the acute phase, and seems to be one explanation for why older TBI patients have poorer outcomes. Additional studies should investigate whether the early identification of acute coagulopathy and prevention of delayed hemostatic perturbation may result in improved morbidity and mortality in aged TBI patients.

## Figures and Tables

**Table 1:** Baseline characteristics of the study population before or after propensity score matching in the Nippon Medical School group.

Variable	Unmatched Subgroups					Matched Subgroups				
	Elderly Subgroup (*n* = 287)		Non-Elderly Subgroup (*n* = 464)		Standardized Mean difference	Elderly Subgroup (*n* = 221)		Non-Elderly Subgroup (*n* = 221)		Standardized Mean Difference
Male, *n* (%)	167	(58.2)	374	(80.6)	−0.512	150	(67.9)	153	(69.2)	−0.022
GCS Score	13	(9–15)	13	(7–14)	0.119	13	(9–15)	13	(8–15)	0.024
ASDH, *n* (%)	197	(68.6)	252	(54.3)	0.305	147	(66.5)	139	(62.9)	0.084
AEDH, *n* (%)	33	(11.5)	78	(16.8)	−0.169	27	(12.2)	24	(10.9)	0.031
TICH, *n* (%)	137	(47.7)	237	(51.1)	−0.060	107	(48.4)	98	(42.1)	0.080
TSAH, *n* (%)	156	(54.4)	298	(64.2)	−0.204	129	(58.4)	132	(59.7)	−0.041
AIS-head	4	(4–5)	4	(3–5)	0.170	4	(4–5)	4	(4–5)	0.013
AIS-face	0	(0–0)	0	(0–0)	−0.333	0	(0–0)	0	(0–0)	0.028
AIS-chest	0	(0–0)	0	(0–3)	−0.414	0	(0–0)	0	(0–0)	−0.024
AIS-abdomen	0	(0–0)	0	(0–0)	−0.333	0	(0–0)	0	(0–0)	0.000
AIS-extremities	0	(0–0)	0	(0–2)	−0.236	0	(0–0)	0	(0–1)	−0.058
AIS-external	0	(0–1)	0	(0–1)	0.021	0	(0–1)	0	(0–1)	0.087
ISS	20	(16–25)	25	(16–29)	−0.288	20	(16–25)	21	(16–26)	−0.042
FFP (mL)	0	(0–0)	0	(0–0)	−0.010	0	(0–0)	0	(0–0)	−0.020

Elderly subgroup, patients aged ≥75 y; non-elderly subgroup, patients aged 16–74 y. All values are expressed as number (%) or median (first to third quartile). AEDH, acute epidermal hematoma; AIS, Abbreviated Injury Score; ASDH, acute subdural hematoma; FFP, fresh frozen plasma; GCS, Glasgow Coma Scale; ISS, injury severity score; TICH, traumatic intracerebral hematoma/contusion; TSAH, traumatic subarachnoid hemorrhage.

**Table 2:** Baseline characteristics of the study population before or after propensity score matching in the Kawaguchi Municipal Medical Center group.

Variable	Unmatched Subgroups					Matched Subgroups				
	Elderly Subgroup (*n* = 108)		Non-Elderly Subgroup (*n* = 435)		Standardized Mean difference	Elderly Subgroup (*n* = 103)		Non-Elderly Subgroup (*n* = 103)		Standardized Mean Difference
Male, *n* (%)	55	(50.9)	330	(75.9)	−0.549	55	(53.4)	55	(53.4)	0.000
GCS Score	8	(4–14)	10	(6–14)	−0.125	8	(4–14)	9	(6–13)	−0.098
ASDH, *n* (%)	85	(78.7)	255	(58.6)	0.413	80	(77.7)	78	(75.7)	0.047
AEDH, *n* (%)	7	(6.5)	92	(21.1)	−0.389	7	(6.8)	6	(5.8)	0.041
TICH, *n* (%)	81	(75.0)	324	(74.5)	0.023	77	(74.8)	80	(77.7)	−0.070
TSAH, *n* (%)	91	(84.3)	357	(82.1)	0.053	86	(83.5)	88	(85.4)	−0.055
AIS-head	4	(4–5)	4	(3–5)	0.282	4	(4–5)	4	(4–5)	−0.056
AIS-face	0	(0–0)	0	(0–0)	−0.234	0	(0–0)	0	(0–0)	−0.077
AIS-chest	0	(0–3)	0	(0–3)	0.025	0	(0–3)	0	(0–3)	−0.080
AIS-abdomen	0	(0–0)	0	(0–0)	−0.137	0	(0–0)	0	(0–0)	−0.051
AIS-extremities	0	(0–2)	0	(0–1)	0.051	0	(0–1)	0	(0–1)	0.025
AIS-external	0	(0–1)	1	(0–1)	−0.176	0	(0–1)	0	(0–1)	−0.080
ISS	25	(18–29)	25	(16–30)	0.116	25	(17–29)	25	(18–33)	−0.097
FFP (mL)	0	(0–770)	0	(0–0)	0.246	0	(0–560)	0	(0–0)	0.080

Elderly subgroup, patients aged ≥75 y; non-elderly subgroup, patients aged 16–74 y. All values are expressed as number (%) or median (first to third quartile). AEDH, acute epidermal hematoma; AIS, abbreviated injury score; ASDH, acute subdural hematoma; FFP, fresh frozen plasma; GCS, Glasgow Coma Scale; ISS, injury severity score; TICH, traumatic intracerebral hematoma/contusion; TSAH, traumatic subarachnoid hemorrhage.

**Table 3:** Glasgow Outcome Scale score at 3 months after injury in elderly group and non-elderly group. Variables were compared using the χ^2^ test.

GOS	Elderly Group (*n* = 324)		Non-Elderly Group (*n* = 324)		*p* Value
4–5, *n* (%)	166	(51.2)	202	(62.3)	0.004
1–3, *n* (%)	158	(48.8)	122	(37.7)	

Elderly group, patients aged ≥75 y; non-elderly group, patients aged 16–74 y. GOS score of 1 indicates death; 2, persistent vegetative state; 3, severe disability; 4, moderate disability; and 5, good recovery.

**Table 4:** Logistic regression analysis of initial variables for independent risk factors for poor prognosis at the time of admission (R^2^ = 0.64).

Factor	Odds Ratio (95% CI)		*p* Value
Age (10-y increments)	1.46	(1.26–1.69)	<0.001
Male	1.02	(0.62–1.68)	0.93
GCS score (1-point decrements)	1.38	(1.29–1.48)	<0.001
AIS-head (1-point increments)	2.42	(1.52–3.86)	<0.001
ISS (1-point increments)	1.09	(1.05–1.12)	<0.001
ASDH	1.85	(0.96–3.57)	0.07
AEDH	0.41	(0.19–0.92)	0.03
TICH	1.69	(1.03–2.79)	0.04
TSAH	1.42	(0.85–2.35)	0.18
PT (0.1-INR increments)	1.01	(0.99–1.02)	0.30
APTT (1-s increments)	1.01	(0.99–1.04)	0.36
Fibrinogen (10-mg/dL decrements)	1.04	(1.01–1.06)	0.001
D-dimer (10-μg/mL increments)	1.08	(1.02–1.13)	0.006

AEDH, acute epidermal hematoma; AIS, Abbreviated Injury Score; APTT, activated partial thromboplastin time; ASDH, acute subdural hematoma; GCS, Glasgow Coma Scale; ISS, injury severity score; PT-INR, prothrombin time-international normalized ratio; TICH, traumatic intracerebral hematoma/contusion; TSAH, traumatic subarachnoid hemorrhage.

## Abbreviations

95% CI	95% confidence interval
AEDH	acute epidural hematoma
AIS	Abbreviated Injury Score
APTT	activated partial thromboplastin time
ASDH	acute subdural hematoma
CT	computed tomography
FFP	fresh frozen plasma
GCS	Glasgow Coma Scale
GOS	Glasgow Outcome Scale
IQR	interquartile range
ISS	Injury Severity Score
MRI	magnetic resonance imaging
PT-INR	prothrombin time-international normalized ratio
TBI	traumatic brain injury
TF	tissue factor
TICH	traumatic intracerebral hematoma/contusion
TSAH	traumatic subarachnoid hemorrhage
α2-PI	α2 plasmin inhibitor
